# Cervical Cancer Screening Access for Women Who Experience Imprisonment in Ontario, Canada

**DOI:** 10.1001/jamanetworkopen.2018.5637

**Published:** 2018-12-07

**Authors:** Fiona G. Kouyoumdjian, Andres McConnon, Emma R. S. Herrington, Kinwah Fung, Aisha Lofters, Stephen W. Hwang

**Affiliations:** 1 Department of Family Medicine, McMaster University, Hamilton, Ontario, Canada; 2 ICES, Toronto, Ontario, Canada; 3 Centre for Urban Health Solutions, St. Michael’s Hospital, Toronto, Ontario, Canada; 4 Faculty of Medicine, McMaster University, Hamilton, Ontario, Canada

## Abstract

**Importance:**

Women who experience imprisonment have higher rates of cervical cancer. Lack of access to cervical cancer screening in the community or in prison may contribute to increased cervical cancer incidence.

**Objectives:**

To determine cervical cancer screening rates for women in provincial prison in Ontario, Canada, and to compare these data with data for the general population.

**Design, Setting, and Participants:**

This retrospective cohort study used correctional and health administrative data from January 1, 2006, to December 31, 2013. Participants included Ontario women aged 21 to 69 years during the follow-up period with no history of cervical cancer or hysterectomy. Analyses were conducted between July 2017 and September 2018.

**Exposures:**

Women admitted to Ontario provincial prison in 2009 or 2010 and released in 2010 were considered exposed, and women in the general population of Ontario were considered unexposed.

**Main Outcomes and Measures:**

Whether women were overdue for cervical cancer screening at the time of admission to prison or on July 1, 2010, for the general population, defined as not having been screened in the previous 3 years, and whether women who were overdue were still overdue after 3 years.

**Results:**

There were 4553 women in the prison group and 3 647 936 women in the general population group. The median (interquartile range) age was 36 (29-43) years in the prison group and 43 (34-53) years in the general population. Women in the prison group had 2.20 times (95% CI, 2.08-2.33) the odds of being overdue for cervical cancer screening compared with women in the general population after adjusting for neighborhood income quintile, at 53.9% (95% CI, 51.8%-56.1%) compared with 32.9% (95% CI, 32.8%-33.0%) (*P* < .001). Women in the prison group also had nearly twice the odds of still being overdue at 3 years, with an odds ratio of 1.87 (95% CI, 1.76-1.99) after adjusting for neighborhood income quintile, and rates of still being overdue of 36.2% (95% CI, 34.5%-38.0%) compared with 21.9% (95% CI, 21.8%-21.9%) (*P* < .001).

**Conclusions and Relevance:**

Women who experience imprisonment have worse cervical cancer screening access than women in the general population. Work should be done to promote cervical cancer screening awareness and to improve access to acceptable screening in prison and in the community after prison release.

## Key Points

**Question:**

Are women who experience imprisonment less likely to be up-to-date on cervical cancer screening than women in the general population?

**Findings:**

In this population-based cohort study, 53.9% of the 4553 women in the prison group were overdue for cervical cancer screening compared with 32.9% of the 3 647 936 women in the general population, which was a significant difference.

**Meaning:**

Efforts should be made to improve access to cervical cancer screening for women who experience imprisonment.

## Teaser

This cohort study compares proportions of women in provincial prison vs women in the general population who were up-to-date for cervical cancer screening between 2006 and 2013 in Ontario, Canada.

## Introduction

In Canada and the United States, research over the last 50 years has shown that women who experience imprisonment are several times more likely to have cervical cancer in their lifetime compared with women in the general population.^1^ Limited evidence suggests that mortality due to cervical cancer is also several times greater in women who experience imprisonment.^2^ This increased burden may be attributable in part to the high prevalence of human papillomavirus risk factors that increase the risk of cervical cancer, such as lifetime number of sexual partners, younger age at first intercourse and first pregnancy, and high parity,^3,4,5,6,7^ as well as other independent risk factors such as smoking and HIV infection.^7^

Another potential contributor to greater cervical cancer burden is access to cervical cancer screening, which is associated with decreased cervical cancer incidence and mortality.^8,9,10^ Several studies using self-reported data suggest that a large proportion of women in prison are up-to-date with Papanicolaou testing, with screening rates of 69% in the past 5 years for 66 women in a UK prison,^8,9,11,12,13,14,15^ 90% in the past 3 years for 133 women in 2 county jails in California in 2002,^16^ and 67% in the past year for 100 women in a prison in Rhode Island in 2008.^17^ In contrast, studies using administrative data in British Columbia, Canada, found that 47% of 209 women in a prison in 1995 had been screened between 1992 and 1994,^18^ 41.1% of 136 women in prison in 2000 and 2002 had been screened in the previous 30 months,^14^ and only 50% of women who participated in a screening intervention were screened in the subsequent 3 years.^19^

There are several limitations to current evidence regarding cervical cancer screening access in women who experience imprisonment. All studies have had small sample sizes and have been conducted in only 1 or 2 facilities,^20^ so findings may be imprecise or not representative of the total population. Self-reported data^20^ may overestimate the frequency of screening,^14,17,18,19,20,21,22,23^ and screening access may differ for women who volunteer to participate in research. Finally, data for most studies were collected more than 10 years ago^14,17,18^ and may not reflect current screening practices.

In this study, we examined the proportion of women released from provincial prison in 2010 who were overdue for cervical cancer screening at the time of admission to prison. We compared the percentage overdue in this population with the general population. We also determined whether women who were overdue for testing on admission to prison accessed testing in the subsequent 3 years. We explored time in prison and primary care encounters during the period under study as potential opportunities for screening.

## Methods

We obtained study approval from the Hamilton Integrated Research Ethics Board. While we accessed nominal data for the purposes of data linkage, we did not obtain informed consent from participants, as the study met criteria for a waiver of consent as articulated in the Canadian Tri-Council Policy Statement on Ethical Conduct for Research Involving Humans,^24^ and the waiver of consent was approved by the Research Ethics Board. This report follows Strengthening the Reporting of Observational Studies in Epidemiology (STROBE) reporting guideline and Reporting of Studies Conducted Using Observational Routinely-Collected Data (RECORD) reporting guidelines.^25^

### Study Design and Setting

We conducted a retrospective cohort study. We compared data on all women released from provincial prison in Ontario, Canada, in 2010 and all women in the general population who were eligible for cervical cancer screening. Provincial correctional facilities in Canada generally house persons who are admitted to prison prior to sentencing or who are sentenced to less than 2 years in prison, as well as persons sentenced to 2 years or longer prior to being transferred to a federal prison and those in temporary detention for other reasons.^26,27,28^ We use the term *provincial prison* to represent all provincial correctional facilities, including jails and prisons. In Ontario, provincial prisons are publicly funded and administered.

For Ontario residents, including Canadian citizens, permanent residents, Indigenous persons, and persons working full-time on a valid work permit for whom Ontario is their primary residence, health services such as hospitalizations, medically necessary surgical procedures, physician services including preventive services such as cancer screening, and medical tests are paid for through the public health insurance system (Ontario Health Insurance Plan [OHIP]), including while persons are in provincial prison.^17,18,19,20^

### Study Cohort and Data Linkage

For the purposes of a separate study, the Ontario Ministry of Community Safety and Correctional Services (MCSCS) provided identifying data on all adults released in 2010 from provincial prison, including name, date of birth, sex, self-reported race, OHIP number, and dates of admission and release and reasons for release between 2005 and 2015. They transferred these data to ICES (formerly the Institute for Clinical Evaluative Sciences), an independent, nonprofit organization funded by the Ontario Ministry of Health and Long-Term Care, which houses health administrative data for Ontario residents. We linked data on persons released from provincial prisons with a unique encoded identifier in the Registered Persons Database, which is a comprehensive database of all persons in Ontario who are eligible for OHIP coverage.^29^ To link data, we used the OHIP number when provided and valid; otherwise, we used a validated deterministic or probabilistic linkage method using name and date of birth.^30^ We excluded linkages that seemed to be incorrect, including the following instances: persons whose date of birth or sex differed between the MCSCS data and the Registered Persons Database, the same unique encoded identifier matched to multiple persons, MCSCS data showed that the person was in custody after the date of death given in the Registered Persons Database, or the person had accessed health care after the date of death in the MCSCS data.

We identified women in the prison group and in the general population who were eligible for cervical cancer screening. We identified women in the general population based on the Registered Persons Database, excluding those released from provincial prison in 2010. We defined the index date as the date of the admission leading to the initial release in 2010 for the prison group, and as July 1, 2010, for the general population group. We included women who were between the ages of 21 and 69 years for the full period under study as this is the eligible age range for cervical cancer screening in Ontario.^31^ Specifically, we included women between the ages of 24 and 66 years on the index date, which meant they would be eligible for cervical cancer screening in the prior 3 years and subsequent 3 years. We excluded women with a history of cervical cancer in the Ontario Cancer Registry or hysterectomy in the Canadian Institute for Health Information Discharge Abstracts Database, as these women would not be eligible for routine screening. For women in the general population, we excluded those who were not eligible for OHIP coverage on July 1, 2010. For women in the prison group, we excluded women who had been transferred to federal prison in the 3 years prior to the index date, as health care data for those persons would not be captured in provincial health databases. We excluded women whose index date was prior to 2009, to maintain comparable periods of study for the prison group and general population controls.

### Variables

#### Covariates

For the prison group, we used data from the MCSCS on time in provincial prison and self-reported race. We included data on race given the known overrepresentation of Indigenous and Black persons in provincial prisons^32^ and the association between race and cervical cancer screening in other jurisdictions.^33^ We maintained the category names provided by the MCSCS, eg, *Aboriginal* for Indigenous persons. No data were available for the general population on race. We determined neighborhood income quintile for the prison release group using address data from the MCSCS, which were the address in the community at the time of admission to provincial prison, and from the Registered Persons Database for the general population. For both groups, we defined sex using data in the Registered Persons Database.

We accessed data in OHIP regarding primary care encounters, which we defined as outpatient visits to general practitioners or family physicians, whether in walk-in clinics or community practices. As physicians working in provincial prison bill OHIP for clinical encounters, primary care encounters in provincial prison would be captured in OHIP, as well as encounters in the community.

#### Outcomes

The primary outcome was whether women were overdue for cervical cancer screening on the index date. We selected a screening interval of 3 years to reflect current guidelines for the frequency of screening in Ontario.^29,34,35^ We categorized women as up-to-date on the index date if they had had a Papanicolaou test in the previous 3 years,^36^ and otherwise we considered women overdue for screening, consistent with language used in Ontario.^33^ We defined cervical cancer screening based on codes in the OHIP database that are billed by physicians who perform Papanicolaou testing or cytopathologists who interpret the test. A secondary outcome was whether women who were overdue on the index date obtained screening over the subsequent 3 years. Any Papanicolaou testing done in provincial prison would be included in these data, as physicians bill OHIP for Papanicolaou testing in provincial prisons.

### Statistical Analysis

For persons in the prison group and general population, we calculated the median and interquartile range (IQR) and percentage in each category (ages 24-29, 30-39, 40-49, 50-59, and 60-66 years) for age, the percentage of persons in each neighborhood income quintile, and the median number of primary care encounters in the 3 years before and 3 years after the index date. For the prison group, we calculated the percentage of persons in each self-reported race category, the percentage with 0, 1, 2, or 3 or more admissions to provincial prison in the 3 years before and 3 years after the index date, the median and IQR for time in provincial prison in the 3 years before and 3 years after the index date, and the median and IQR for time in provincial prison in the 3 years prior to and 3 years after the index date.

We calculated the percentage and 95% confidence interval of women in the prison group and general population who were overdue for screening on the index date. Of those who were overdue, we calculated the proportion who obtained screening in the subsequent 3 years. We used χ^2^ tests to compare the prison group and the general population in terms of the proportion overdue, and we considered the result significant at the level of 2-sided *P* < .05.

For the prison group compared with the general population, we used unadjusted logistic regression to estimate odds ratios (ORs) and 95% confidence intervals for being overdue on the index date and for still being overdue at 3 years. We adjusted the models for neighborhood income quintile as a potential confounder of the association between imprisonment status and cervical cancer screening.

For persons in the prison group who were overdue on the index date and who were still overdue at 3 years, we calculated the proportion with any primary care encounters and any primary care encounters in provincial prison, the number of primary care encounters, and the time in provincial prison in the 3 years before and 3 years after the index date.

## Results

Of 53 955 persons released from provincial prisons in 2010, 52 546 (97.4%) were successfully linked with health administrative data (eFigure in the Supplement). We excluded those released for 1 day or less in 2010 (n = 233), those not eligible for cervical cancer screening because of male sex (n = 45 956), age (n = 1429), prior cervical cancer (n = 7) or hysterectomy (n = 280), admission prior to 2009 (n = 13), and time in federal prison prior to the index date (n = 75). We included the remaining 4553 women in the study as the prison group.

We identified 4 948 430 women in the general population who were aged 24 to 66 years on July 1, 2010. We excluded women in the prison group (n = 5617) and women who were not eligible for OHIP coverage on July 1, 2010 (n = 907 435), as well as women with a history of cervical cancer (n = 6972) or hysterectomy (n = 380 470). We included the remaining 3 647 936 women as the general population.

Women in the prison group were younger (median [IQR] age, 36 [29-43] years) compared with those in the general population (median [IQR] age, 43 [34-53] years), as shown in Table 1. Sixty-one percent of women in the prison group were in the lowest 2 neighborhood income quintiles, compared with 38.4% of women in the general population. A high proportion of women in the prison group self-reported Aboriginal (n = 659 [14.5%]) or Black (n = 296 [6.5%]) race.

**Table 1.** Sociodemographic Characteristics of Cervical Cancer Screen–Eligible Women Who Were Released From Provincial Prison in Ontario, Canada, in 2010 and in the General Population

Characteristic^a^	Prison Group (n = 4553)	General Population (n = 3 647 936)
Age, median (IQR), y	36 (29-43)	43 (34-53)
Age group, No. (%)		
24-29 y	1240 (27.2)	531 411 (14.6)
30-39 y	1628 (35.8)	921 128 (25.3)
40-49 y	1285 (28.2)	986 696 (27.0)
50-59 y	345 (7.6)	800 503 (21.9)
60-66 y	55 (1.2)	408 198 (11.2)
Neighborhood income quintile, No. (%)		
Missing	307 (6.7)	24 112 (0.7)
1	1838 (40.4)	688 831 (18.9)
2	936 (20.6)	710 581 (19.5)
3	620 (13.6)	726 284 (19.9)
4	488 (10.7)	762 181 (20.9)
5	364 (8.0)	735 947 (20.2)
Self-reported race, No. (%)		
Aboriginal	659 (14.5)	NR
Black	296 (6.5)	NR
Other	963 (21.2)	NR
White	2635 (57.9)	NR
Time in provincial prison, median (IQR), d		
3 y prior to index date	0 (0-21)	NA
3 y after index date	22 (4-90)	NA
Provincial prison admissions, No. (%)		
3 y prior to index date		
0	2531 (55.6)	NA
1	722 (15.9)	NA
2	423 (9.3)	NA
≥3	877 (19.3)	NA
3 y after index date		
0	2346 (51.5)	NA
1	856 (18.8)	NA
2	501 (11.0)	NA
≥3	850 (18.7)	NA
Primary care encounters		
3 y prior to index date		
Any, No. (%)	3794 (83.3)	3 288 901 (90.2)
Median (IQR), No.	11 (3-25)	9 (4-16)
3 y after index date		
Any, No. (%)	4027 (88.4)	3 241 294 (88.9)
Median (IQR), No.	12 (3-30)	8 (3-15)

Abbreviations: IQR, interquartile range; NA, not applicable; NR, not reported.

^a^ Characteristics on index date, which is the date of admission leading to first release from provincial prison in 2010 for the prison group or July 1, 2010, for the general population.

Of the 4553 women in the prison group, 53.9% (95% CI, 51.8%-56.1%) (n = 2454) were overdue for cervical cancer screening on the index date and 46.1% (n = 2099) were up-to-date. Of the 2454 women who were overdue on the index date, 67.2% (n = 1648) were not screened and 32.8% (n = 806) were screened over the subsequent 3 years.

Women in the prison group were more likely to be overdue for cervical cancer screening on the index date compared with the general population (53.9% [95% CI, 51.8%-56.1%] vs 32.9% [95% CI, 32.8%-33.0%]; *P* < .001), as shown in the Figure and Table 2. The unadjusted OR was 2.38 (95% CI, 2.25-2.53) for the prison group compared with the general population. The OR decreased to 2.20 (95% CI, 2.08-2.33) after adjusting for neighborhood income quintile.

**Figure.**
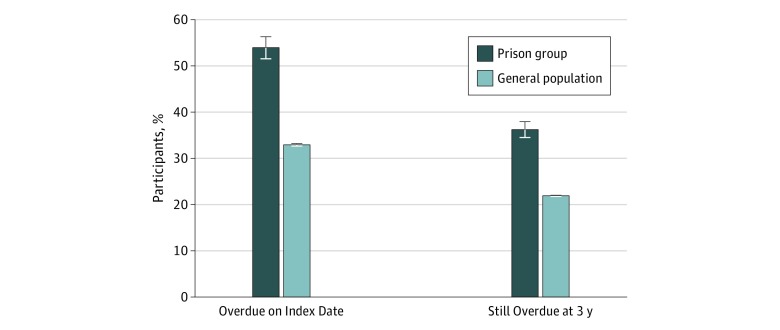
Cervical Cancer Screening Status of Women Released From Provincial Prison and the General Population in Ontario, Canada The prison population included 4553 women and the general population included 3 647 936 women. The index date is the date of admission to prison leading to first release in 2010 for the prison group and July 1, 2010, for the general population. Error bars indicate 95% confidence intervals.

**Table 2.** Cervical Cancer Screening Status for Women Released From Provincial Prison in Ontario, Canada, in 2010 and the General Population

Screening Status	Prison Group (n = 4553)	General Population (n = 3 647 936)
Overdue on index date^a^		
No. (%) [95% CI, %]	2454 (53.9) [51.8-56.1]	1 200 169 (32.9) [32.8-33.0]
Unadjusted OR (95% CI)	2.38 (2.25-2.53)	1 [Reference]
Adjusted OR^b^ (95% CI)	2.20 (2.08-2.33)	1 [Reference]
Still overdue at 3 y after index date^a^		
No. (%) [95% CI, %]	1648 (36.2) [34.5-38.0]	798 272 (21.9) [21.8-21.9]
Unadjusted OR (95% CI)	2.03 (1.91-2.16)	1 [Reference]
Adjusted OR^b^ (95% CI)	1.87 (1.76-1.99)	1 [Reference]

Abbreviation: OR, odds ratio.

^a^ Index date is the date of admission to prison leading to first release in 2010 for the prison group and July 1, 2010 for the general population.

^b^ Adjusted for neighborhood income quintile.

By 3 years after the index date, 36.2% (95% CI, 34.5%-38.0%) of all women in the prison group were still overdue for cervical cancer screening, compared with 21.9% (95% CI, 21.8%-21.9%) of women in the general population (*P* < .001) (Figure and Table 2). The OR for still being overdue was 2.03 (95% CI, 1.91-2.16) without any adjustment and 1.87 (95% CI, 1.76-1.99) after adjusting for neighborhood income quintile.

Of the 2099 women in the prison group who were up-to-date at the time of admission, 11.7% (n = 246) had accessed a Papanicolaou test while in provincial prison in the 3 years prior to the index date. For the 806 women who were overdue on the index date and accessed screening in the subsequent 3 years, 24.2% (n = 195) accessed a Papanicolaou test while in provincial prison during the 3 years after the index date.

In the prison group, most women who were overdue for screening had multiple primary care encounters over the follow-up period (Table 3). Of women who were overdue on the index date, 73.7% had had any primary care encounters, with a median (IQR) of 6 (0-18) encounters in the previous 3 years, and 27.8% had had any primary care encounters in provincial prison in the previous 3 years. Of those who were still overdue at 3 years, 67.3% had had primary care encounters in the 3 years prior to the index date and 79.4% had primary care encounters in the 3 years after the index date, with a majority having had multiple encounters and more than half having had any encounters in provincial prison (Table 3).

**Table 3.** Primary Care Encounters and Time in Prison for Cervical Cancer Screen–Eligible Women Released From Provincial Prison in Ontario, Canada, in 2010, by Cervical Cancer Screening Status

Experiences of Prison Group	Overdue on Index Date^a^ (n = 2454)	Still Overdue at 3 y After Index Date^a^ (n = 1648)
Primary care encounters		
3 y prior to index date		
Any, No. (%)	1809 (73.7)	1109 (67.3)
Median (IQR), No.	6 (0-18)	4 (0-15)
Any in prison, No. (%)	683 (27.8)	413 (25.1)
3 y after index date		
Any, No. (%)	2068 (84.3)	1308 (79.4)
Median (IQR), No.	9 (2-25)	5 (1-18)
Any in prison, No. (%)	1448 (59.0)	918 (55.7)
Time in provincial prison, No. (%)		
3 y prior to index date, d		
Any	1097 (44.7)	689 (41.8)
1-6	295 (12.0)	189 (11.5)
7-29	273 (11.1)	168 (10.2)
30-89	249 (10.1)	166 (10.1)
90-179	172 (7.0)	106 (6.4)
≥180	108 (4.4)	60 (3.6)
3 y after index date, d		
1-6	760 (31.0)	552 (33.5)
7-29	543 (22.1)	388 (23.5)
30-89	527 (21.5)	360 (21.8)
90-179	322 (13.1)	193 (11.7)
≥180	302 (12.3)	155 (9.4)

Abbreviation: IQR, interquartile range.

^a^ Index date is the date of admission to prison leading to first release in 2010 for the prison group.

Of the 2454 women in the prison group who were overdue for screening on the index date, 44.7% had been in provincial prison in the previous 3 years and 21.5% had spent 30 days or longer in provincial prison during that period: 10.1% for 30 to 89 days, 7.0% for 90 to 179 days, and 4.4% for 180 days or more (Table 3). Of the 1648 women who were still overdue for screening at 3 years after the index date, 42.9% spent 30 or more days in provincial prison in the 3 years after the index date: 21.8% for 30 to 89 days, 11.7% for 90 to 179 days, and 9.4% for 180 days or more.

## Discussion

In this population-based study, we found that even in the context of a universal health insurance system, more than half of women released from provincial prisons in Ontario in 2010 were overdue for Papanicolaou testing at the time of prison admission. This proportion is significantly higher than the proportion of women in the general population who were overdue, which was 32.9% (*P* < .001). Three years later, 36.2% of all women in the prison group had still not received any cervical cancer screening, compared with 21.9% of women in the general population (*P* < .001). Most women in the prison group who were not screened had had multiple primary care encounters during this period, and many spent a substantial amount of time in provincial prison.

In this study, the proportion of women in prison whose cervical cancer screening status was up-to-date was lower than the proportion found in US and UK studies using self-reported data,^24^ but very similar to the results of older studies from British Columbia, Canada, using administrative data.^25^ Our findings are also consistent with research from Ontario that identifies lower cervical cancer screening rates in other marginalized populations,^14,17,18^ including groups that are overrepresented in provincial prisons such as persons with low income.

### Limitations

There are several potential limitations to this study. We may have underestimated Papanicolaou testing if women accessed testing in hospital, outside of Ontario, in federal prison, or on a First Nation during the period under study, or if physicians did not bill OHIP for Papanicolaou testing. This type of bias may be differential across groups, and may have increased the apparent difference between groups; however, we think these issues are relatively uncommon and therefore we expect that this would not substantially affect our results. These data reflect the screening status of women between 2008 and 2010, and over the subsequent 3 years. Since then, Ontario guidance changed regarding the routine screening interval and when to initiate screening,^19,20^ and the province implemented a program to send letters to the public regarding cervical cancer screening. While the proportion of persons accessing screening may have changed since these data were collected, we expect the difference in screening rates likely persists between people in prison and the general population because no specific policy or program has been implemented in provincial prison. Health administrative data tell us only whether women obtain testing and not whether they want testing or whether they are being offered testing; research is needed to elucidate these issues. In this study we were not trying to establish a causal association between imprisonment history and lack of cervical cancer screening. In this setting, there is an overrepresentation of persons with other known risk factors for cervical cancer screening, including persons with low socioeconomic status and persons who are Indigenous and Black, which may explain the high proportion of persons overdue in this setting. Our study nonetheless reveals that a focus on this population and setting is indicated as part of cervical cancer prevention.

## Conclusions

This study shows a lack of access to cervical cancer screening in women who experience provincial imprisonment. In several studies, women in prison have indicated that they would be willing to access Papanicolaou testing in prison,^37,38,39^ and in a study in Brazil, a majority of women had accessed testing in prison and a majority of those who had not accessed testing in prison specified that lack of opportunity was the reason why they didn’t access testing in prison.^33^ Barriers to testing for women in prison may include a lack of access to acceptable health care in prison^14,18,23^ and on release,^40^ and a lack of knowledge regarding what a Papanicolaou test is and why this testing is done.^21,41^ Another issue may be that given the high prevalence of comorbidity in this population,^21^ the attention of patients and health care professionals is focused on specific conditions at the cost of other health conditions and preventive care.^18,22,23^

Based on the findings of this study, we suggest several opportunities to address the inequity in cervical cancer screening access. Health promotion activities could support women in prison understanding the indications for and methods of Papanicolaou testing,^42,43^ for example, through providing group sessions on sexual health.^44^ Because women in prisons have often experienced child abuse and intimate partner violence,^22,45,46^ health care services, including cervical cancer screening, should be trauma informed.^46^ Innovative strategies such as self-collection swabs for human papillomavirus instead of routine Papanicolaou testing could support access and minimize harms without sacrificing accuracy.^47^ More broadly, work should be done to strengthen health care in prison and to support continuity of care for women on release. Specific measures could include documenting cervical cancer screening status in health records for follow-up and communication with community health care professionals, as well as linkage on release with primary care^34,37^ and organized screening programs,^9,41,48^ if indicated and desired.

## Notes

Supplement. **eFigure.** Flow Diagram for Data Linkage and Definition of Prison Group and General Population Group of Women Eligible for Cervical Cancer Screening
