# Supplementary material for: Cervical Cancer Screening Access for Women Who Experience Imprisonment in Ontario, Canada
Source: JAMA Netw Open. 2018 Dec 7;1(8):e185637. doi: 10.1001/jamanetworkopen.2018.5637 (PMC6324332; doi:10.1001/jamanetworkopen.2018.5637)
Supplement: Supplement. — eFigure. Flow Diagram for Data Linkage and Definition of Prison Group and General Population Group of Women Eligible for Cervical Cancer Screening [file jamanetwopen-1-e185637-s001.pdf]

Supplementary Online Content

Kouyoumdjian FG, McConnon A, Herrington ERS, Fung K, Lofters A, Hwang SW. Cervical cancer screening access for women who experience imprisonment in Ontario, Canada. *JAMA Netw Open*. 2018;1(8):e185637. doi:10.1001/jamanetworkopen.2018.5637

eFigure. Flow Diagram for Data Linkage and Definition of Prison Group and General Population Group of Women Eligible for Cervical Cancer Screening

This supplementary material has been provided by the authors to give readers additional information about their work.

© 2018 Kouyoumdjian FG et al. *JAMA Network Open.*

eFigure. Flow diagram for data linkage and definition of prison group and general population group of women eligible for cervical cancer screening

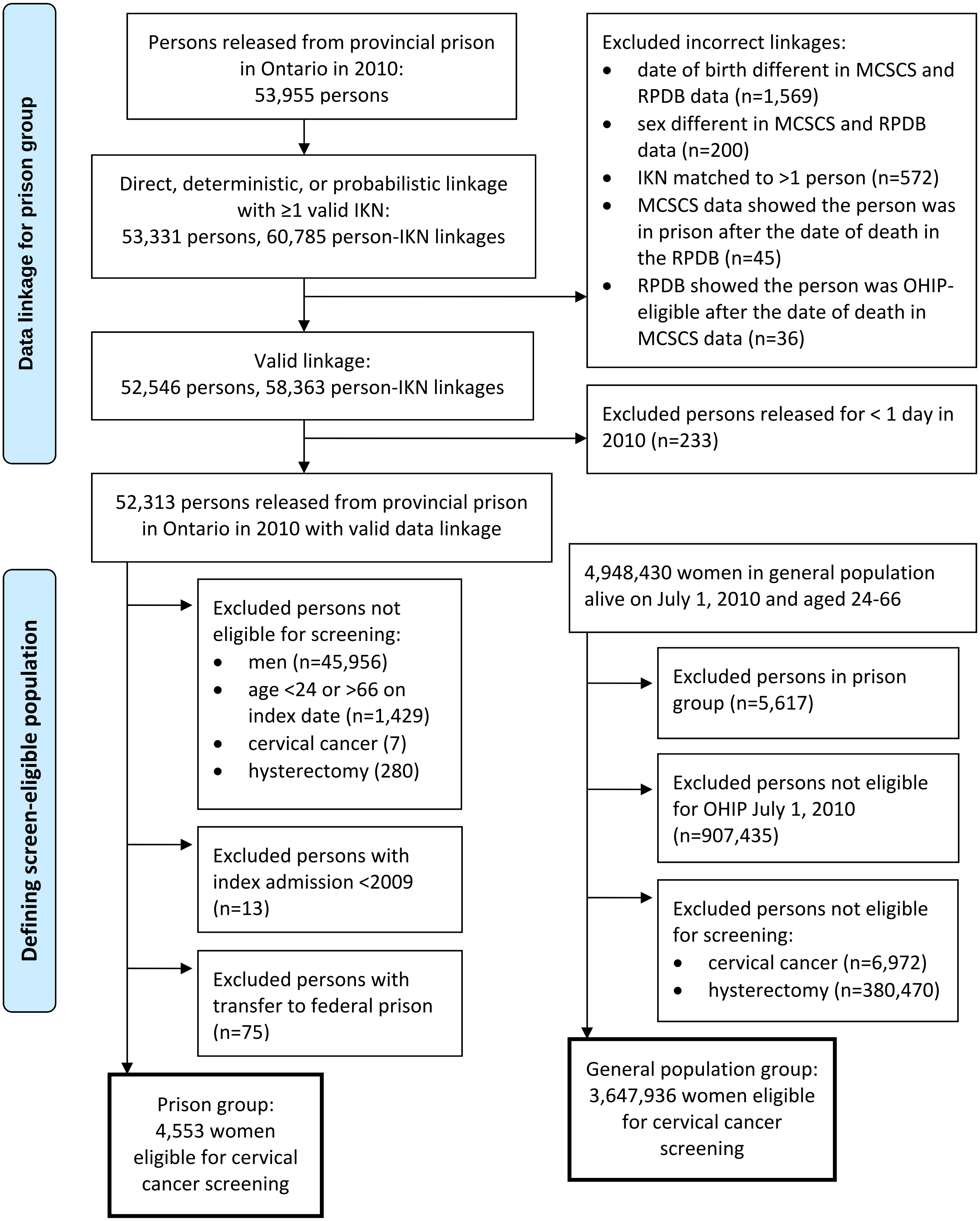

© 2018 Kouyoumdjian FG et al. *JAMA Network Open.*
